# Hair Coil Penile Tourniquet Syndrome in an Unusual Age

**DOI:** 10.1155/2015/642547

**Published:** 2015-02-11

**Authors:** Kursad Zengin, Mustafa Yasar Ozdamar, Sebahattin Albayrak, Serhat Tanik, Muhittin Atar, Hasan Bakirtas, Muhammed Abdurrahim Imamoglu, Mesut Gurdal

**Affiliations:** ^1^Department of Urology, Faculty of Medicine, Bozok Universitesi Uygulama ve Arastirma Hastanesi, Uroloji AD, 66100 Yozgat, Turkey; ^2^Department of Pediatric Surgery, Faculty of Medicine, Bozok Universitesi Uygulama ve Arastirma Hastanesi, Uroloji AD, 66100 Yozgat, Turkey

## Abstract

Penile tourniquet syndrome (PTS), a rare urologic emergency, may lead to undesirable results including necrosis and amputation of penis, if not diagnosed and treated appropriately. Sometimes these injuries may be accepted as a forensic case. Miscellaneous objects used for strangulation can be metallic or nonmetallic. Of all ages, the most vulnerable period is infancy. Telogen effluvium is the most common cause of PTS in infants who are 0–6 years old. In the literature, telogen effluvium as a reason of PTS was not found except for this age group. Therefore, we aimed to present a boy who is 8 years old diagnosed as PTS because of his mother's hair coil.

## 1. Introduction

Penile tourniquet syndrome (PTS) is usually caused by a hair coil wrapped around the sulcus coronarius of penis. The complications range from simple edema to necrosis. When we reviewed the literature, between the years of 1967 and 2014, it was seen that the cause of penile strangulation in babies was mother's hair, with its specific nomenclature telogen effluvium. It was significantly encountered in circumcised boys. Urethrocutaneous fistula, complete urethral transection, penile gangrene, or penile amputations were presented among the reported complications in the literature [1, 2].

Hair coil penile tourniquet syndrome is usually seen in boys between 0 and 6 years old [1]. Herein, we aimed to report an experience with the diagnosis and treatment of an 8-year-old boy with a complicated hair coil penile strangulation.

## 2. Case Presentation 

**Figure 1:**
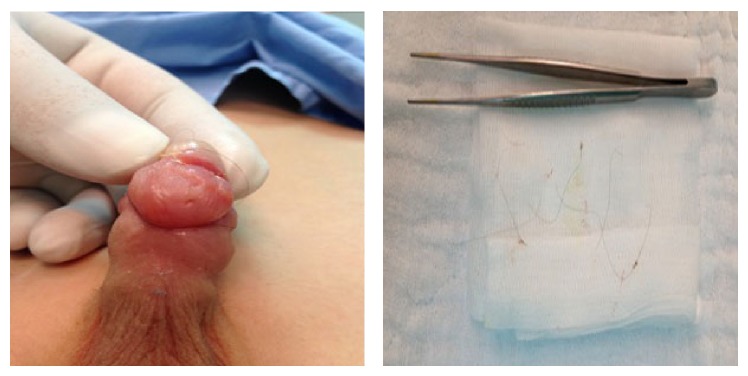
The appearance of penile tourniquet syndrome at the time of diagnosis, and causative factor: hair strand of mother.

The patient's mother recognized an increasing swelling in glans penis and sulcus coronarius for 24 hours in her 8-year-old boy. They were admitted to urology clinics of a medical center. They were prescribed local antibiotics. After 24 hours of this treatment, the swelling increased, and they were admitted to our outpatient clinic. In the physical examination, a hair strand in the sulcus coronarius which was coiled around penile shaft and another island-like region on the ventral side of glans penis was found (Figure 1). It was also noticed that the patient had an obvious glob vesical.

**Figure 2:**
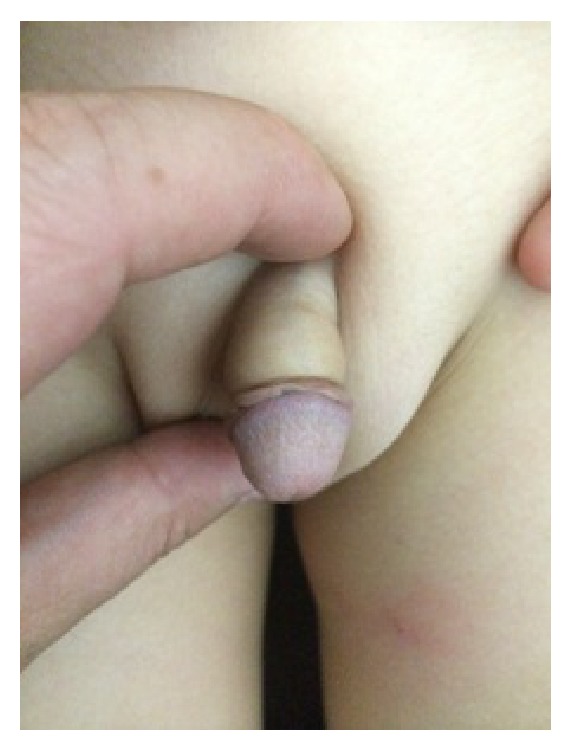
The appearance of penis at the third week.

The patient also complained about voiding difficulty. Under general anesthesia the hair was cut and excised. Debridement around sulcus was performed and a healthy urethra was noticed. Peroral antibiotics and anti-inflammatory medication were initiated after the operation. The patient can urinate without any instrumentation postoperatively. Patient was discharged on the postoperative second day. His parents were advised once a week for follow-up visit. On the postoperative third week, complete recovery was observed (Figure 2).

## 3. Discussion

PTS commonly occurs in the appendicular organs of infants like genitalia, toes, and fingers. The most common cause is hair coil injury with 79% of incidences [1, 3]. Although PTS is mostly encountered in infants of mothers with postpartum excessive hair loss (telogen effluvium) [3], our patient was an 8-year-old boy with PTS. In the literature, we did not find such a case in this age.

It was previously postulated that if the localization of PTS was the coronal sulcus and distal part of it, the perpetrator may be either the hair of mother (telogen effluvium) which was accidental or the deliberate cases because of enuresis or urinary incontinence [2].

In the PTS, penile injury, respectively, starts with penile edema and glandular deterioration, followed by superficial penile injuries, urethral fistula, and even complete urethral transection. Therefore, if the embedded hair in penis was not recognized on physical examination in the early stage, severe complications might occur [4].

When a patient administered with penile symptoms, which demonstrated demarcation line and edema, PTS should be suspected. He should be carefully examined under anesthesia in terms of PTS. This will prevent misdiagnosis and malpractice [4]. We diagnosed PTS in our patient through a careful examination and treated it with removal of the hair coil followed by debridement of the necrotic tissue under general anesthesia in 10 minutes.

PTS usually occurs in infants but can be seen in older boys such as our patient. These cases may be accidental or intentional. Parents may punish their children due to bedwetting and strive to hide the intent. Barton et al. declared a case of PTS because of child abuse [5].

PTS is a serious condition especially in infants but can be prevented with the education of parents. On the other hand, when the symptoms of circular penile injury, edema, and sharp demarcation line are observed, PTS should be thought in the differential diagnosis. PTS is an emergency condition that should be intervened immediately. Additionally, PTS might be a forensic case especially when child abuse, misdiagnosis, and malpractice were suspected.
